# Pooled sputum to optimise the efficiency and utility of rapid, point-of-care molecular SARS-CoV-2 testing

**DOI:** 10.1186/s12879-021-06316-z

**Published:** 2021-07-08

**Authors:** Alison Burdett, Christofer Toumazou, Rashmita Sahoo, Adam Mujan, Tsz-Kin Hon, Judith Bedzo-Nutakor, Nicola Casali, Maria Karvela, Mohammadreza Sohbati, Graham S. Cooke, Gary W. Davies, Luke S. P. Moore

**Affiliations:** 1 DnaNudge Ltd, Imperial College White City Campus, The Translation and Innovation Hub, Level 11, 84 Wood Lane, London, W12 0BZ UK; 2 Department of Electrical and Electronic Engineering, Imperial College London, Exhibition Road, London, SW7 2AZ UK; 3 North West London Pathology, Imperial College Healthcare NHS Trust, Fulham Palace Road, London, W6 8RF UK; 4 NIHR Health Protection Research Unit in Healthcare Associated Infections & Antimicrobial Resistance, Imperial College London, Du Cane Road, London, W12 0NN UK; 5 Chelsea and Westminster NHS Foundation Trust, 369 Fulham Road, London, SW10 9NH UK

**Keywords:** COVID-19, Coronavirus, Molecular diagnostics, PCR

## Abstract

**Background:**

As SARS-CoV-2 testing expands, particularly to widespread asymptomatic testing, high sensitivity point-of-care PCR platforms may optimise potential benefits from pooling multiple patients’ samples.

**Method:**

We tested patients and asymptomatic citizens for SARS-CoV-2, exploring the efficiency and utility of CovidNudge (i) for detection in individuals’ sputum (compared to nasopharyngeal swabs), (ii) for detection in pooled sputum samples, and (iii) by modelling roll out scenarios for pooled sputum testing.

**Results:**

Across 295 paired samples, we find no difference (*p* = 0.1236) in signal strength for sputum (mean amplified replicates (MAR) 25.2, standard deviation (SD) 14.2, range 0–60) compared to nasopharyngeal swabs (MAR 27.8, SD 12.4, range 6–56). At 10-sample pool size we find some drop in absolute strength of signal (individual sputum MAR 42.1, SD 11.8, range 13–60 vs. pooled sputum MAR 25.3, SD 14.6, range 1–54; *p* < 0.0001), but only marginal drop in sensitivity (51/53,96%). We determine a limit of detection of 250 copies/ml for an individual test, rising only four-fold to 1000copies/ml for a 10-sample pool. We find optimal pooled testing efficiency to be a 12–3-1-sample model, yet as prevalence increases, pool size should decrease; at 5% prevalence to maintain a 75% probability of negative first test, 5-sample pools are optimal.

**Conclusion:**

We describe for the first time the use of sequentially dipped sputum samples for rapid pooled point of care SARS-CoV-2 PCR testing. The potential to screen asymptomatic cohorts rapidly, at the point-of-care, with PCR, offers the potential to quickly identify and isolate positive individuals within a population “bubble”.

**Supplementary Information:**

The online version contains supplementary material available at 10.1186/s12879-021-06316-z.

## Background

Molecular testing, predominantly reverse transcriptase polymerase chain reaction (RT-PCR), remains the standard of care for detection of SARS-CoV-2 due to high sensitivity and specificity. However laboratory-based RT-PCR requires significant infrastructure, is often centralised, and turnaround times frequently exceed 24 h. This has driven supplementary testing with rapid lateral flow antigen tests, but these often exhibit sub-optimal sensitivity [1, 2]. Developing molecular diagnostic tests for use outside of laboratory settings, with retained ‘gold standard’ test performance characteristics, could accelerate clinical decision making, enabling effective triage and infection control measures in frontline clinical and community settings.

The CovidNudge assay potentially meets this need, as a point-of-care, real-time RT-PCR test without the need for significant laboratory facilities or sample preparation, with a turnaround time of 90 min. The platform comprises a single-use DnaCartridge and a processing unit (the NudgeBox) with six viral targets (*RdRp-IP2*, *RdRp-IP4*, *e*-gene, *n1*, *n2*, *n3*) and one host gene as a sample adequacy control (Ribonuclease P, *RNaseP*) (supplementary Fig. 1a, supplementary Table 1), generating positive, negative, or indeterminate results (supplementary Table 2) [3]. The sensitivity of this assay compared with laboratory-based PCR using nasopharyngeal swab samples was found to be 97% (95% CI 89.6–99.6) with a specificity of 100% (95% CI 98.5–100) [3].

As the need for COVID-19 testing continues to develop, particularly for asymptomatic screening, near-patient rapid testing must further respond. Currently each NudgeBox processes one sample at a time, and although an increase in throughput can be accommodated by deploying additional platforms, the use of pooled patient samples may enable incremental throughput [4]. The USA Food and Drug Administration (FDA) currently recommends two approaches to specimen pooling [5]; pooled aliquots of transport media each containing a single patient sample (sample pooling), or combined swabs from multiple patients into a single volume of transport media (swab pooling). UK guidelines currently only recommend sample pooling, as swab pooling provides no mechanism to deconvolve individual positive samples [6]. So far, pooling has not been widely used in clinical or community settings. For laboratory-based molecular platforms this is likely because of; a lack of validated pooling techniques; delayed reporting of positive pools from subsequent individual sample retesting; and difficulties in fine resolution identification of appropriate low-prevalence cohorts to deploy in.

For point-of-care RT-PCR platforms, potential benefits from pooling may be more easily realised. Near-patient tests enable a positive pool to be quickly followed up with individual testing, while a negative pool enables rapid de-escalation of restrictions. However, neither of the pooling methods described earlier are compatible with the CovidNudge platform, as the system has no requirement for liquid transport media, therefore no simple method exists for combining swabs from individual patients. As an alternative, sputum can be directly expectorated without the use of a swab, with comparable sensitivity and specificity to oro- or nasopharyngeal samples in previous analyses [7]. Although sputum is considered a difficult sample to process with traditional RT-PCR platforms as the high viscosity poses challenges in transfer with a fixed volume transfer bulb, these limitations do not occur on the CovidNudge platform since a sample can be directly transferred to the DnaCartridge using an appropriate swab.

This study explores the efficiency and utility of CovidNudge among symptomatic [8] patients and asymptomatic citizens (i) for detection of SARS-CoV-2 in individuals’ sputum (in comparison to nasopharyngeal swabs), (ii) for detection of SARS-CoV-2 in pooled sputum, and (iii) by modelling roll out scenarios for pooled sputum testing.

## Method

### Sputum sampling

To investigate whether sputum samples are compatible with the CovidNudge platform, we undertook a comparative analysis of nasopharyngeal swab samples with sputum. Testing took place in September and October 2020 using samples from two separate groups: patients admitted to hospital via the emergency department at Chelsea & Westminster NHS Foundation Trust, and asymptomatic members of the London Symphony Orchestra. Testing of emergency admissions at Chelsea & Westminster NHS Foundation Trust was done as a service evaluation approved by the Trust COVID-19 Testing Committee. All participants consented to supplying a contemporaneous nasopharyngeal swab and sputum sample (supplementary method).

Sputum samples were collected into a sample tube with inactivating agent (Oragene500, DNAgenotek) [9]. Nasopharyngeal samples were tested on the CovidNudge platform; from hospitalised patients at the point of care as part of normal clinical practice; from asymptomatic members of the London Symphony Orchestra at the point of collection (Covent Garden, London). Sputum samples from both hospitalised patients and orchestra members were tested in laboratory facilities at DnaNudge premises (Imperial College Translation and Innovation Hub, Wood Lane, London). All sputum samples were opened, sampled, and inserted into the DnaCartridge within a biosafety cabinet (NUARE: Class II 12469:2000, Model No: NU-543-300S) following Health and Safety Executive (HSE) requirements relating to handling of samples from persons with respiratory illnesses. To test the sputum samples, an RNA/DNA buccal swab (SK-2, Isohelix) was used, rubbed in the inactivated sputum for 5 seconds, then inserted into the DnaNudge cartridge (supplementary Fig. 1b). The cartridge was then inserted into the NudgeBox and a test run following standard procedure [3].

To determine whether the number of replicates amplifying during the CovidNudge test could serve as a semi-quantitative marker for viral load, for the positive samples we correlated the number of replicates amplifying on the CovidNudge platform against cycle threshold values obtained using laboratory PCR platforms (supplementary method).

### Sputum pooling

Pools were tested with one positive sample and the rest as negative samples, using a single buccal swab to sequentially “dip” the sputum samples. Initial exploratory analysis was performed starting at a pool of two and then incrementing the pool by adding further negative samples each time, up to a maximum pool size of 40 and 30. In practice these pool sizes are unlikely to be practical unless prevalence is very low, therefore the characteristics of pooling were further elucidated using a pool size *n* = 10. UK guidelines currently recommend a pool size between 6 and 12 [6]. USA FDA guidelines regarding the validation of n-pooled tests recommend that samples from at least 20 positive patients and (20 x n) negative patients should be collected and tested with one positive and (n – 1) negative samples per pool [5]. For our validation we took a larger number of positive samples (*n* = 51) to allow us to vary the position of the positive sample within the pool during the test (i.e. positive sample in first position, second position,... ninth position, tenth position) at least 3 times. The pooled samples were dipped in turn with a single Isohelix swab (supplementary method), then inserted into a DnaCartridge and tested following standard procedure [3]. Negative samples dipped subsequent to the positive patient sample were discarded as contaminated; negative samples dipped prior to the positive sample were retained for reuse. Positive samples were resampled a maximum of three times, as each sampling introduced dilution from addition of negative sputum into the positive sample.

### Limits of detection for pooling

A negative sputum sample was spiked with viral genetic material (Microbiologics HE0062S process control pellet, Lot: HE0062–01, Expiry: 2022-01-31) dissolved in molecular water. The viral solution was serially diluted and aliquots of 25uL were added to a 25uL sputum sample. These sequentially diluted samples were then absorbed onto an Isohelix swab and run on the CovidNudge platform using established methods [3] to determine the limit of detection (LOD).

### Optimising pooling size

The efficiency gained through pooling of samples is highly dependent on the prevalence of positive patients in the cohort to be tested. We modelled pooling efficiency as a function of prevalence, considering two scenarios:

- Single pooled test: if the pooled test is reported as positive, the patient samples are all individually tested to determine which of the samples is positive
- Nested pooled test: if the pooled test is positive, smaller patient pools are repeated to narrow down the search for the positive result(s).

To explore the relative efficiency of single versus nested pooling, we simulated the result of different pooling scenarios (supplementary method). We selected a range of single and nested pooling scenarios, with a maximum initial pool size of 12 as per UK guidelines [6]. We also limited the total number of nested testing cycles to three (i.e. one or two rounds of pooling with a final individual testing round) to minimise delays to results which would detract from the benefit of having a rapid RT-PCR test at the point of care. For each initial pool size, all possible input sample permutations were evaluated. For a pool size of *n*, this results in *2*^*n*^ input sample vectors. Each input vector was evaluated in turn to determine the total number of tests that would need to be run in the given pooling scenario. Finally, the probability of the specific input vector being realised was calculated as a function of prevalence.

### Statistical analysis

Descriptive statistics were used, with chi squared tests for categorical variables, t-test for normally distributed continuous data (normal distribution was determined using the Kolmogorov-Smirnov test) and Mann-Whitney U test for non-parametric data. Pearson’s correlation was used as a measure of strength of association when comparing sample type and semi-quantification.

## Results

### Sputum sampling

Paired nasopharyngeal and sputum samples were obtained from symptomatic [8] and asymptomatic hospital patients admitted through the emergency department diagnosed as SARS-CoV-2 positive (*n* = 74) or negative (*n* = 103), and asymptomatic screening from members of the London Symphony Orchestra (*n* = 118). 295 paired samples were obtained.

**Fig. 1:**
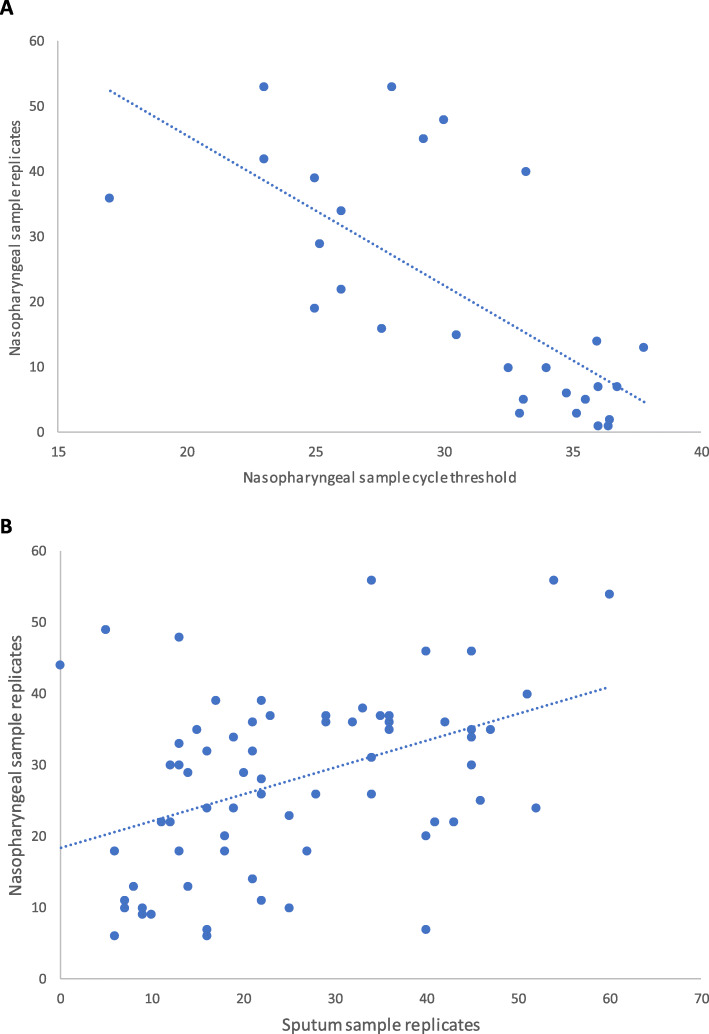
Comparison of CovidNudge amplified replicates (1**A**) versus laboratory RT-PCR cycle threshold, and (1**B**) for nasopharyngeal swabs versus sputum samples

First, for the 74 positive patient samples we determined whether the number of replicates amplifying during the CovidNudge test can serve as a semi-quantitative marker for viral load when compared to cycle threshold values from laboratory RT-PCR platforms (Pearson’s correlation coefficient, r = − 0.71, *p* < 0.001, Fig. 1A). Second, comparing this semi-quantitative measure of positivity between paired nasopharyngeal and sputum samples, we find them comparable (Table 1, Fig. 1B). The mean number of replicates amplifying for sputum was 25.2 (standard deviation (SD) 14.2, range 0–60) and for nasopharyngeal was 27.8 (SD 12.4, range 6–56). Both sets of data were shown to be normally distributed (sputum samples: D = 0.126, *p* = 0.183; nasopharyngeal samples: D = 0.065, *p* = 0.899) and not significantly different − 2.6 (95% CI = − 5.93 to 0.73, *p* = 0.124).

**Table 1:** Nasopharyngeal and sputum paired samples tested on the CovidNudge platform, London, 2020

		SPUTUM SAMPLES	
		POSITIVE	NEGATIVE
NASOPHARYNGEAL SAMPLES	POSITIVE	73	1
	NEGATIVE	0	221

Legend: Sputum samples demonstrated 98.65% sensitivity (95% CI = 92.7–99.97%) and 100% specificity (95% CI = 98.3–100%) against nasopharyngeal samples

Three outlying results were identified with high number of nasopharyngeal replicates but low replicates in sputum samples (Fig. 1B). Two results had at least some detectable sputum replicates (nasopharyngeal 49, sputum 5, and nasopharyngeal 48, sputum 13), these two patients had 4 days of illness prior to swabbing; one had well controlled diabetes mellitus and the other was at the extreme of old age. One result had no detectable sputum replicates (nasopharyngeal 44, sputum 0), this patient was day 14 of illness and had been admitted for a non-COVID related illness and had no respiratory symptoms at the time of illness.

### Sputum pooling

At the extreme end of the iterative pool-size ranging exercise, the platform was able to report a positive result from a single positive sputum sample in a pool of 40 and was repeatable in 3/5 pools (3/5 detected > 13 replicates, 2/5 detected only 2 replicates and were therefore defined as indeterminate).

**Fig. 2:**
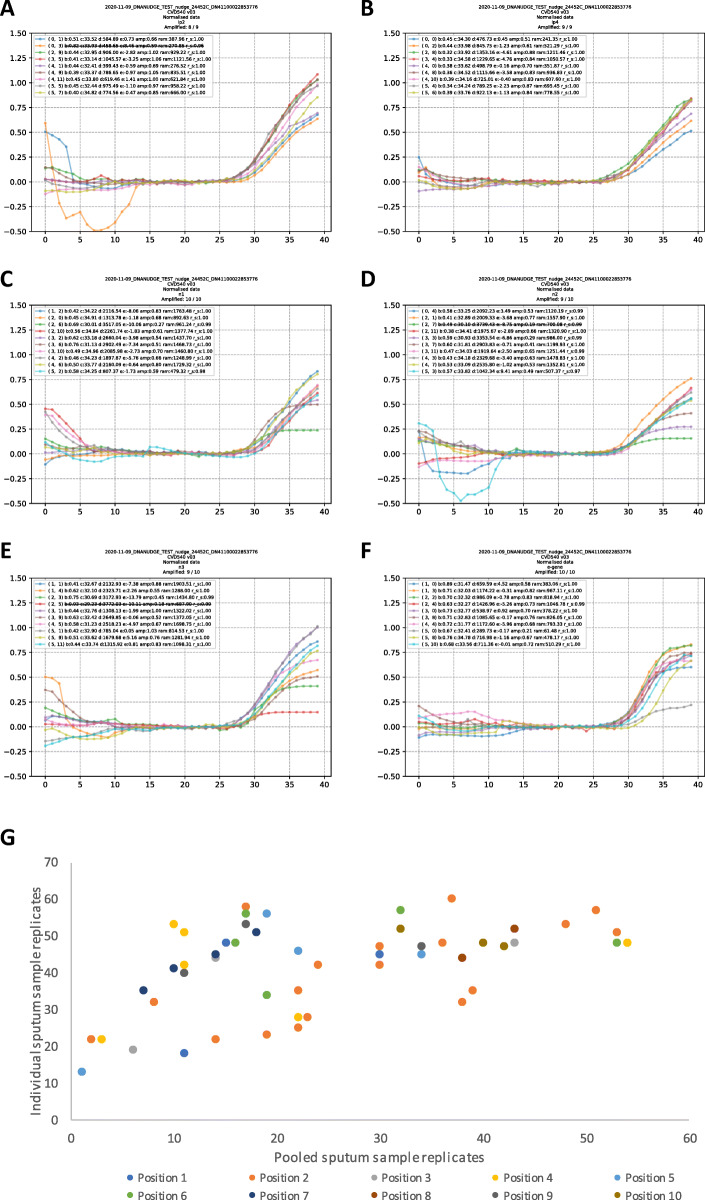
CovidNudge PCR amplification curves for a pool of 1 positive and 9 negative samples across all six gene targets (2**A** = RdRp-IP2, 2**B** = RdRp-IP4, 2**C** = n1, 2**D** = n2, 2**E** = n3, 2**F** = e-gene), and with variation of the position of the positive across the 10-pool (2**G**). Legend: 2**A**-**F**. X-axis PCR cycle, Y-axis replicate well threshold. All 6 viral target genes showed amplification in most replicates. 2**G**. Coloured dots denote how testing the positive sputum sample in a pool of 10 varied when the positive sample altered position in relation to the negatives (from position 1–10)

To evaluate the more functional 10-pool testing, 53 pools were run with a single positive and 9 negative samples (example run in Fig. 2A-F) to determine sensitivity, and 200 negative samples in all-negative pools to determine specificity (Table 2). The mean replicates amplifying for individual sputum samples was 42.1 (SD 11.8, range 13–60) and for pooled sputum samples was 25.3 (SD 14.6, range 1–54). Both sets of data were shown to be normally distributed (single sputum samples: D = 0.168, *p* = 0.090; pooled sputum samples: D = 0.122, *p* = 0.381), with a significant difference between single and pooled sputum replicates 16.8 (95% CI = 11.7 to 21.9, *p* < 0.0001).

**Table 2:** Test performance characteristics for pooled sputum samples on the CovidNudge platform, London, 2020

		POOLED SAMPLES		
		POSITIVE	INDETERMINATE	NEGATIVE
INDIVIDUAL SAMPLES	POSITIVE	51	2	0
	INDETERMINATE	0	0	0
	NEGATIVE	0	0	200

Legend: 53 patients with positive individual samples were tested with 200 negative results. Pools were either tested as 10 negative samples, or one positive sample with 9 negative samples. An indeterminate result is reported when only one or two replicates amplify, i.e. the signal is at the limits of detection. Pooled samples demonstrated 96.23% sensitivity (95% CI = 87.0–99.5) and 100% specificity (95% CI = 98.2–100) against individual samples

The ‘position’ of the positive sample in the pool (from first to tenth) was evaluated, with the positive sample placed in each position among the pool sampling 3 times (Fig. 2G). This demonstrated no significant correlation (r = 0.06, *p* = 0.68) between sample position and number of replicates.

### Limits of detection for pooling

For individual sample testing the LOD was measured as 250 copies per swab; this climbed to 1000 copies per swab for 10 pool testing. The 4-fold increase in LOD for a 10-pool test suggests dipping a swab into successive sputum samples provides less sample dilution than the expected n-fold reduction in viral concentration for an n-pool test.

### Putting pooling into practice

**Fig. 3:**
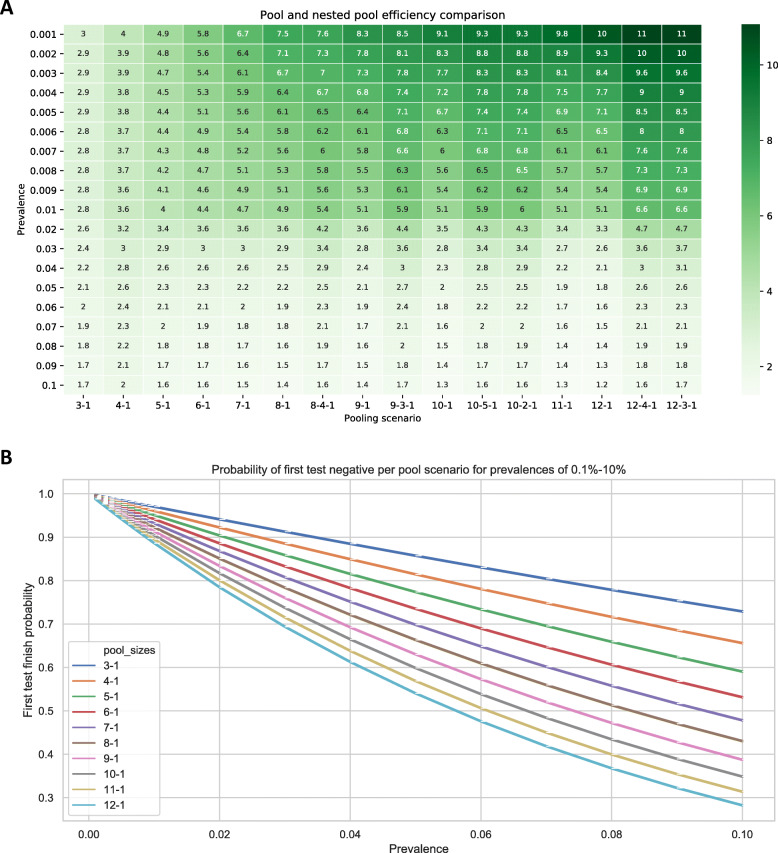
Relative testing efficiency for various pool-size strategies for CovidNudge sputum PCR testing (3**A**) across climbing COVID-19 prevalence with (3**B**) probability of a negative first pool. Legend: 3**A**. Calculation of the relative efficiency for different pooling strategies as a function of prevalence, where relative efficiency (*Er*) is defined as $\mathrm{Er}\kern0.5em =\frac{\mathrm{Number}\kern0.5em \mathrm{test}\mathrm{s}\kern0.5em \mathrm{required}\kern0.5em \mathrm{with}\mathrm{out}\kern0.5em \mathrm{pooling}}{\mathrm{Average}\kern0.5em \mathrm{number}\kern0.5em \mathrm{of}\kern0.5em \mathrm{test}\kern0.5em \mathrm{required}\kern0.5em \mathrm{with}\kern0.5em \mathrm{pooling}\kern0.5em }$ 3**B**. For prevalence below 2%, pooling of up to 12 samples has a high probability (> 75%) of returning a negative result of first test. As prevalence increases, to maintain high efficiency of pooling, the pool size should decrease. At 5% prevalence, to maintain a 75% probability of a negative first test, the pool size should decrease to 5

For the scenarios simulated, all pooling strategies have an efficiency (i.e. ratio of number of tests required without pooling, compared to mean number of tests required with pooling) greater than 1 for prevalence up to 10% (Fig. 3A & B). In all cases simulated, the nested pooling scenario results in a higher efficiency than the simple pooling case; however the trade-off is a longer wait for the final confirmatory result in case of a positive result in one or more of the pooled samples.

## Discussion

The CovidNudge system provides rapid near-patient diagnostic capability for SARS-CoV-2 testing. To expand the utility of this platform, we find high levels of concordance in test performance characteristics between nasopharyngeal sampling and sputum. Equally importantly we find pooling of sputum for testing on the CovidNudge platform has no drop in sensitivity, and only marginal fall in the limits of detection. These two aspects together offer the opportunity to optimise the efficiency of novel diagnostic pathways for both patients and for citizens who may need to work or mix in close proximity.

While pooling of samples has the potential to offer significant efficiency gains in terms of consumables, the balance of time-to-result needs to be carefully considered for each potential use-setting. There are now examples of pooling in emerging COVID-19 outbreak scenarios [10, 11] and pooling of sputum for surveillance has already been advocated [12], and initial evaluations undertaken [13]. However for direct clinical care it is imperative to know the definitive result for a patient as soon as possible, and preferably at the point of care (e.g. when planning for an emergency surgery or to optimise utilisation of side room capacity) [14]. In such settings, the requirement for immediacy of results may rule out the use of laboratory based pooling because of the risk that a second round of testing may be required. However there are specific use cases where near-patient pooling (such as we present here) is desirable, particularly when testing asymptomatic population groups. Practical use cases include regular screening of population cohorts (“bubbles”) that are in regular and close contact either inside or outside of normal household groups such as; care home staff, residents, and visitors; family and support networks; workplaces, theatres and concerts, sports teams; school and university classroom groups; healthcare workers. Other researchers have identified the efficiency benefits of sample pooling at the point of care particularly in environments where test resources are scarce [15, 16], and the method described here warrants further investigation to ascertain utility in these settings.

Our sputum sample validation has demonstrated for the first time that a single inactivated sputum sample can be dipped multiple times, without requirements for pipetting small volumes needed in other evaluations of pooling [10, 11, 13, 17], which is impractical at the point of care. Our single pool-dipped swab can then be inserted directly into the DnaCartridge for processing due to the unique swab-to-result nature of the platform. Before n-pool testing, each sputum sample can be individually dipped with a swab and these swabs stored for later individual testing, should the pool test return a positive result.

To ensure accurate resulting for pooled samples, in the current DnaNudge platform, a pooled sample is registered using the multiple patient identifiers of each patient in the pool. Following a pooled negative result, an individual negative result is generated for each patient for onwards reporting into hospital records. A pooled positive result does not generate a result for all patients but instead flags the requirement for immediate re-testing of each individual. This avoids patients who may be negative within a positive pool being erroneously reported for public health follow-up.

Our study has several limitations. First, although the validation of sputum samples against nasopharyngeal samples included both symptomatic and asymptomatic patients, in asymptomatic patients the terminology “sputum” may be somewhat misleading since the samples are unlikely to come from the lower respiratory tract. However, in all but one of the cases, the oral (salivary) sample provided did produce a positive result for SARS-CoV-2 for patients who had tested positive through nasopharyngeal sampling, as found elsewhere [18]. Second, whilst we correlated the number of positive CovidNudge replicates to laboratory-based PCR cycle threshold as a semi-quantitative measure, this is not a true representation of viral load, and this could impact our measurement of pooling to be able to detect low-positive patients in a large pool. To mitigate this we did undertake formal limit of detection analysis, as noted, and our finding of a four-fold fall in LOD in a 10 pool, while not large, must be considered in the context of how pooling is being deployed. Third, while single-patient use of the CovidNudge is a low-skilled activity, enabling use of the platform in a variety of near-patient and community scenarios, the multi-stepped process of pooling would require training, and dedicated cohorts of trained personnel should be used to support this aspect of its use. Finally, it is important to be cognizant with all COVID-19 diagnostics of the need to consider (and test for) variants of concern, and potentially other coronaviridae [19], and the CovidNudge gene targets will be continually evaluated for their utility and changed where needed.

## Conclusions

We describe for the first time the use of sequentially dipped sputum samples using a commercially available buccal swab for rapid near-patient pooled SARS-CoV-2 RT-PCR testing. The sequential dipping of sputum minimises loss of sensitivity through dilution, while also retaining individual samples should a positive test result require further individual testing. The potential to screen asymptomatic cohorts rapidly, at the point of care, with the superior test performance characteristics of RT-PCR (compared to lateral flow devices) [1, 2] offers the potential to quickly identify and isolate positive individuals within a population “bubble”, including in family units, workplaces, and social gatherings.

## Supplementary Information

**Additional file 1.**

## Data Availability

The datasets analysed during the current are available from the lead author (AB alison.burdett@dnanudge.com) on reasonable request, as long as this meets local ethics and research governance criteria.

## Abbreviations

CE mark: Conformité Européene mark
COVID-19: Coronavirus disease 2019
DNA: Deoxyribonucleic acid
*Er*: Relative efficiency
FDA: Food and drug administration
HSE: Health and safety executive
LOD: Limit of detection
RdRp: RNA-dependent RNA polymerase
RT-PCR: Reverse transcriptase polymerase chain reaction
RNA: Ribonucleic acid
SARS-CoV-2: Severe acute respiratory syndrome coronavirus 2
SD: Standard deviation
UK: United Kingdom
USA: United States of America
