# Supplementary material for: Pooled sputum to optimise the efficiency and utility of rapid, point-of-care molecular SARS-CoV-2 testing
Source: BMC Infect Dis. 2021 Jul 8;21:665. doi: 10.1186/s12879-021-06316-z (PMC8265726; doi:10.1186/s12879-021-06316-z)
Supplement: Supplementary file 1 — Additional file 1. [file 12879_2021_6316_MOESM1_ESM.docx]

**Supplementary method**

***Sputum Sample Testing***

**Equipment Required**

- Oragene 500 sample collection tube (DNAgenotek)
- SK-2 buccal swab with tube (Isohelix)

Sputum samples should only be collected by a healthcare professional trained in the technique wearing appropriate PPE. Sputum samples should only be tested by a professional trained in the technique and using appropriate biocontainment measures.

**Sample collection**

1. Prepare the patient for the procedure by asking them to sit upright, rinse their mouth with water and spit out prior to sputum collection.
2. Ask the patient to take a few deep breaths to help loosen secretions; please note, if patient is on a nebuliser, give nebuliser first and wait 10 minutes before taking a sample.
3. The patient should cover their mouth before forcing out a deep cough to release the sputum. Sputum should be collected in the sample tube provided. Ideally the sputum sample should be no less than the size of a small fingernail.
4. Check the quality of the sputum to ensure **it is not** simply saliva, but rather sputum (mixture of phlegm and mucous). If the patient is unable to provide any sputum, advise to keep hydrated where possible, and encourage deep breathing to try again in an hour.
5. Hold the sample tube upright in one hand and close the funnel lid with the other hand firmly until a loud click is heard. The liquid in the lid will be released into the tube to mix with the sputum.
6. Holding the tube upright, unscrew the funnel and discard funnel as clinical waste. Use the small screw cap to close sample tube tightly. Shake the capped tube for 5 seconds.

**Sample Testing:**

1. Unscrew the cap from the sputum sample tube.
2. Remove the cap from Isohelix Swab while retaining the bung with stopper.
3. Mix the swab in the sputum, rubbing gently for 10 seconds to get a good sputum sample on the swab. When extracting the swab from the specimen pot, remove any excess sputum residue by wiping the swab gently against the inside of the pot.
4. Remove the cap from the DnaCartridge and insert the swab end at a vertical angle into the Cartridge (the DnaCartridge cap can be discarded).
5. Press the Isohelix cap with stopper into the Cartridge and gently remove tail of swab, this will leave swab in chamber on removal.
6. Discard the swab tail in a “sharps” bin.
7. Lock the DnaCartridge closed with the Isohelix bung and test as per standard procedure

***Pooled experiments using known sputum samples***

We initially repeated five 10-pool tests using a paediatric flocked swab recommended for routine use for individual nasopharyngeal sampling of patients on the DnaNudge platform (MFS-96000BQ, Shenzhen Medico Technology Company Ltd, LOT: 20200607, Expiry: 2022-06-11). In all five cases, the 10-pool test result was returned as negative. We then switched to using the Isohelix buccal swab which is patterned with a matrix designed to efficiently collect and ‘trap’ buccal cell samples for DNA testing. This patterned matrix has the effect of securely capturing and holding the sputum samples, and so each additional pooled sample simply adds more sputum material onto the swab matrix. The Isohelix swab was then taken forward for use in the experiments described in the study.

**Equipment required:**

- Oragene 500 sample collection tubes (DNAgenotek)
- SK-2 buccal swabs with tube (Isohelix)

Sputum samples should only be tested by a professional trained in the technique and using appropriate biocontainment measures.

**Method**

1. Obtain a number (up to 10) of individual sputum samples following steps 1. – 6. in Appendix 1. Samples should be labelled as positive or negative.
2. Position all sputum sample tubes in a line using a test tube rack or similar receptacle holder and unscrew the lids of each of the tubes.
3. Remove cap from Isohelix swab retaining the bung with stopper, and gently dip the swab in the first sputum sample for 5 seconds, performing 2 gentle rubs for each side of the swab against the sputum and the inside of the tube. Remove any excess sputum hanging off the swab by rubbing the swab gently against the inside of the tube.
4. Once this is done for the first sample, insert the same swab into the next and repeat the process for all 10 samples.
5. Load the Isohelix swab into the DnaCartridge following previously established method [2]
6. Any negative samples that have been dipped with the Isohelix swab after the swab has been dipped into a positive sample must be discarded as contaminated.

***Pooled sputum testing using blinded samples***

**Equipment required:**

- Oragene 500 sample collection tubes (DNAgenotek)
- SK-2 buccal swabs with tube (Isohelix)

**Method**

1. Obtain a number (up to 10) of individual sputum samples following steps 1. – 6. in Appendix
2. Position all sputum sample tubes in a line using a test tube rack or similar receptacle holder and unscrew the lids of each of the tubes.
3. Take a sample of sputum from sample 1 using an Isohelix swab following step 3 in Appendix 2. Replace the cap on the Isohelix swab, label the swab as sample 1, and place to one side for later processing if needed.
4. Repeat step 3 using a new swab each time for all other samples in the pool.
5. Once the individual samples are obtained, using a clean swab, dip the swab in the first sample following step 3 in Appendix 2.
6. Insert the same swab into the next sample; repeat the sampling process for all 10 samples.
7. Load the Isohelix swab into the DnaCartridge following steps 7. – 13. in Appendix 1.

**Interpretation of Results**

- If the pool test result is returned as negative, all samples are negative. The individual samples and swabs can be disposed of.
- If the pool test result is returned as positive, the individual stored swabs are tested in turn to determine which sample(s) in the pool are positive. The sputum samples in the collection tubes should be disposed of.
- If the pooled test result is returned as indeterminate or void, the 10 sputum samples can be re-tested as a pool

**Correlating semi-quantitative CovidNudge and laboratory PCR results**

To confirm that the number of replicates amplifying during the CovidNudge test can serve as a semi-quantitative marker for viral load, we compared the number of positive replicates amplifying on the DnaNudge platform against cycle threshold values obtained using laboratory PCR platforms.

1. Nasopharyngeal samples and sputum samples were collected as noted above.
2. Nasopharyngeal samples and sputum samples were run on CovidNudge and the number of replicates (as a total number across all gene targets; see supplementary table 1) documented.
3. Concomitant nasopharyngeal samples and sputum samples were run on laboratory PCR platforms, and the cycle threshold documented. (the laboratory used a range of platforms, and samples were run on one of: Roche Lightcycler LC96; Thermo Fisher TaqPath; Roche 6800; Abbot M2000 depending on platform capacity and access to consumables at various stages of the pandemic)
4. Statistical correlation between CovidNudge nasopharyngeal sample replicates and laboratory PCR nasopharyngeal cycle threshold was undertaken.
5. Statistical correlation between CovidNudge nasopharyngeal sample replicates and CovidNudge sputum sample replicates was undertaken.

***Calculating optimal pooling size***

We investigated the efficiency of pooling as a function of prevalence, to define the optimal pool size for testing.

1. We define the probability *P(pos)* of returning a positive result from a pooled test is calculated as:

$$P\left( pos \right)=\left( 1-\left( 1-p \right)^{n} \right)$$

where *p* = prevalence and *n* = pool size.

1. We denote a standard “Dorfman” nested pooled test [ref] as (*n_1_* | *n_2_* | *n_3_* …. | *n_x_* ), where *n_1_* > *n_2_* > *n_3_* … > *n_x_*, and where *n_1_* denotes the size of the first pool, *n_2_, n_3_* etc. are the sizes of subsequent sub-pools, and *n_x_* = 1. So a pooling strategy of ( 12 | 3 | 1 ) would start with an initial pool size of 12, followed by 4 pooled tests of 3 samples each, followed by individual testing of any of the n = 3 sub-pools that had tested positive.
2. Using this this nomenclature, the single pooled test can be considered as a nested pool of ( n | 1 ), i.e. if the initial n-pool tests positive, the next round of testing would test all n samples individually.
3. We selected a range of single and nested pooling scenarios, with a maximum initial pool size of 12, but limited the total number of nested testing cycles to three.
4. For each initial pool size, all possible input sample permutations were evaluated. For a pool size of *n*, this results in *2^n^* input sample vectors.
5. Each input vector was evaluated in turn to determine the total number of tests that would need to be run in the given pooling scenario.
6. The probability of the specific input vector being realised was calculated as a function of COVID-19 prevalence, in the range 0.1% through to 10%.

**Supplementary figures and tables**

**Supplementary Figure 1a: NudgeBox (28 × 15*·*5 × 13*·*5 cm; 5 kg) and DnaCartridge (25 × 78 × 85 mm; 40 g)**


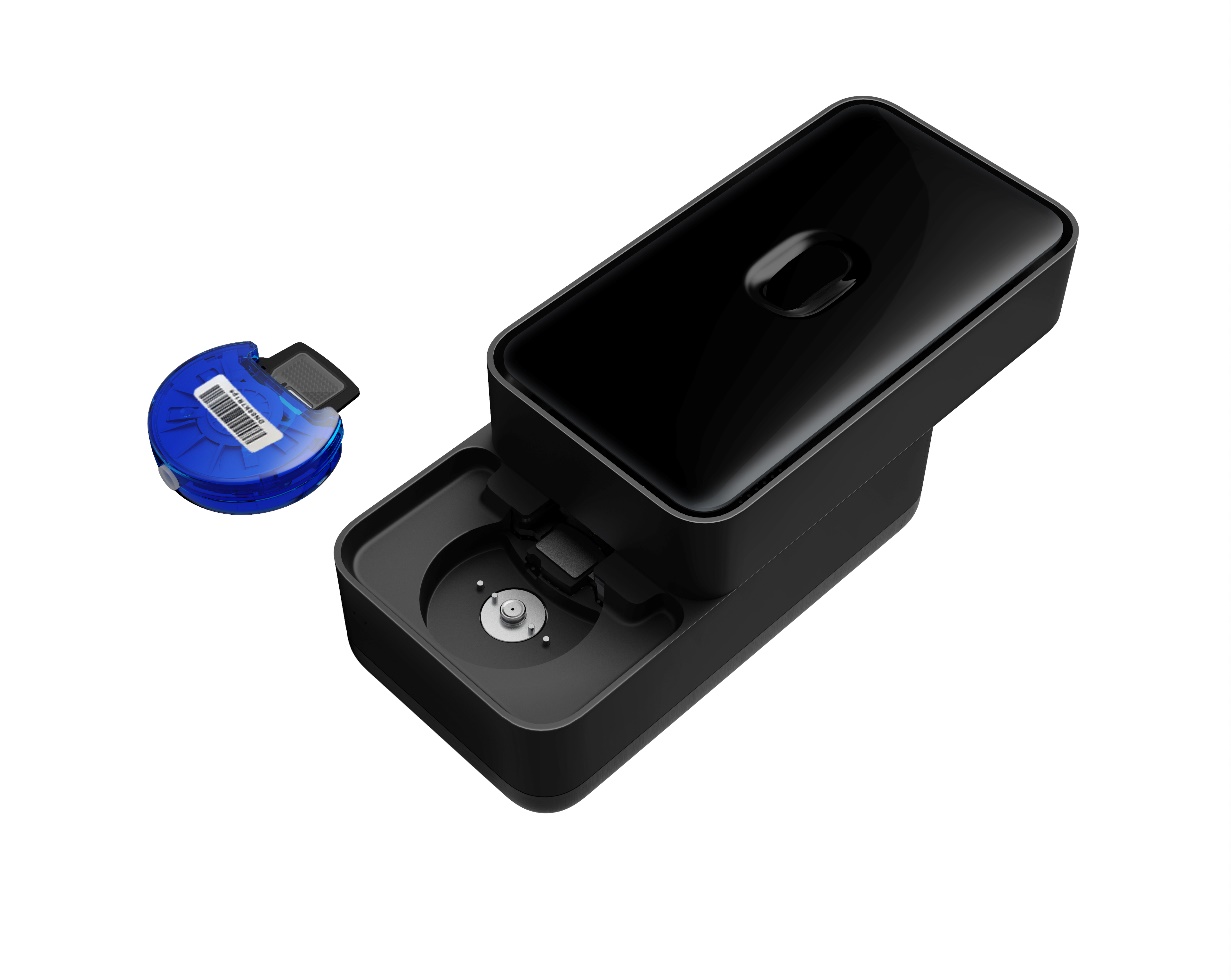


**Supplementary Figure 1b: DnaCartridge with Isohelix swab (with stopper and bung) inserted into the sample chamber**


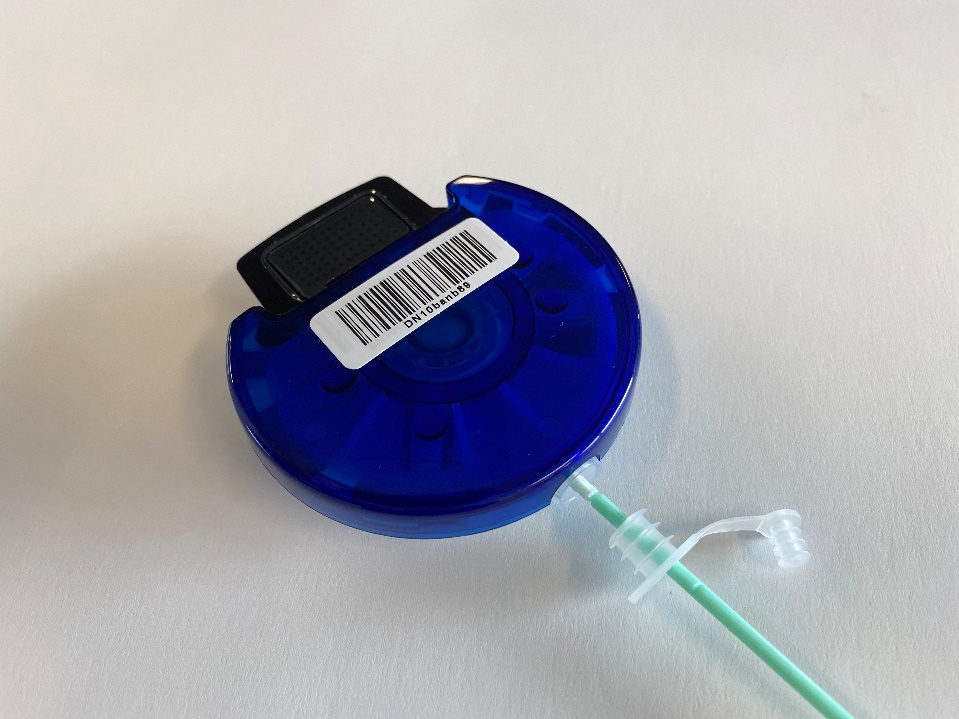


**Supplementary Table 1: CovidNudge gene targets and number of replicates**

| **Gene target** | **Number of replicates** |
| --- | --- |
| n1 | 10 |
| n2 | 10 |
| n3 | 10 |
| RdRP-IP2 | 9 |
| RdRP-IP4 | 9 |
| e | 9 |
| RNAseP (control) | 6 |

**Supplementary Table 2: CovidNudge test result report wording**

| Invalid | Less than 2 of the 6 human RNAseP replicates have amplified. Viral results are not applicable |
| --- | --- |
| Positive | At least 3 of the viral gene replicates have amplified. This could be 3 from the same gene type, or 3 from distinct genes. At least 2 human RNAseP replicates have amplified. |
| Negative | None of the viral targets have amplified. At least 2 human RNAseP replicates have amplified. |
| Indeterminate | One or two of the viral targets have amplified. At least 2 human RNASeP replicates have amplified. |
| Error | A technical issue (insufficient pressure) was detected during the test. The test was aborted. |
